# Analyzing Policies Through a DOHaD Lens: What Can We Learn?

**DOI:** 10.3390/ijerph15122906

**Published:** 2018-12-19

**Authors:** Julia M. Goodman, Janne Boone-Heinonen, Dawn M. Richardson, Sarah B. Andrea, Lynne C. Messer

**Affiliations:** Oregon Health & Science University—Portland State University School of Public Health, Portland, OR 97201, USA; boonej@ohsu.edu (J.B.-H.); dawn.richardson@pdx.edu (D.M.R.); andreasa@ohsu.edu (S.B.A.); lymesser@pdx.edu (L.C.M.)

**Keywords:** DOHaD, policy analysis, birth outcomes, social determinants of health, life course, intergenerational

## Abstract

Social, health, and environmental policies are critical tools for providing the conditions needed for healthy populations. However, current policy analyses fall short of capturing their full potential impacts across the life course and from generation to generation. We argue that the field of Developmental Origins of Health and Disease (DOHaD), a conceptual and research framework positing that early life experiences significantly affect health trajectories across the lifespan and into future generations, provides an important lens through which to analyze social policies. To illustrate this point, we synthesized evidence related to policies from three domains—family leave, nutrition, and housing—to examine the health implications for multiple generations. We selected these policy domains because they represent increasing distance from a reproductive health focus, each with a growing evidence base to support a potential impact on pregnant women and their offspring. Each of these examples represents an opportunity to extend our understanding of policy impact using a DOHaD lens, taking into account the potential life course and intergenerational effects that have previously been overlooked.

## 1. Introduction

Developmental Origins of Health and Disease (DOHaD) is a conceptual and research framework positing that “the state of health and risk from disease later in childhood and adult life is significantly affected by environmental factors acting during the pre-conceptional, prenatal, and/or early postnatal periods” [1]. Central to this description is the prominent role of “the environment” as critical for shaping human health across the lifespan and into future generations. “Environment” encompasses the social, economic, and physical characteristics of the places where we live, work, and play, and these characteristics are products of a vast set of policy decisions. While we have come to understand that policies influence our health in myriad ways, current policy analyses fall short of capturing their full potential impacts, which include intergenerational effects.

Mounting evidence supports the notion that social policies—policies that influence social or economic outcomes but do not necessarily target health directly—have significant public health implications [2,3]. One recent review concluded that most policies relevant to housing/ neighborhoods, marriage/family strengthening, employment, and income supplementation had beneficial mental and/or physical health effects [4]; in other words, positive health outcomes have resulted from policies and interventions not directly designed to impact health. One concrete and well-established example of unintended health consequences resulting from a social policy or intervention is the Moving to Opportunity for Fair Housing Demonstration Project, a housing mobility program intended to increase low-income families’ access to economic opportunity and safer neighborhoods. This multi-city, randomized controlled trail, considered to be one of the most rigorous mobility interventions in the U.S., examined how receipt of a housing voucher impacted families across three different conditions: the experimental group was intended to move to low-poverty neighborhoods, an intermediate group was able to move to any neighborhood, and the control group continued residence in public housing. More than a decade later, randomization to the experimental group was associated with decreased risk of severe obesity and type 2 diabetes, increased physical activity, and improved mental health and well-being for adults [5,6,7], along with worsened mental health, binge drinking, and substance use among boys [8,9].

This body of literature illuminates the often unintended health-related consequences of selected social policies. However, analyses of health effects typically focus on the near-term, where the outcomes are measured within the range of one to 15 years following exposure to the policy. Studies at the far end of that range, like evaluations of Moving to Opportunity, deepen our understanding of the full impacts of policies, including how policy effects may fade over time. However, assessments of these health impacts almost universally remain within a single generation.

DOHaD research suggests that early life exposures have consequences throughout the life course and across generations. Initial work in this field pointed to the role of prenatal undernutrition, often resulting in low birthweight (LBW), in permanently shaping the offspring’s structure, function, and metabolism [10,11]. More recent evidence suggests that this programming may also affect the risk of chronic diseases, including type 2 diabetes [12] and some cancers [13] both of which are influenced by early environmental conditions, and provides insights into how these environmental factors affect how our genes are regulated, altering biological functions and disease processes throughout the life cycle, and passed from generation to generation [14].

Of particular relevance to policy discussions, emerging evidence supports the epigenetic influence of maternal stress [15]. Exposure to a range of stimuli, from job loss to interpersonal discrimination, can trigger a cascade of biological processes producing physiological changes [16]. Repeated and prolonged activation of these processes leads to dysregulation of one’s stress response system, placing individuals at greater risk of depression, cardiovascular disease, and more [16]. In the context of pregnancy, the maternal stress response system and that of the developing fetus are intrinsically linked. The dysregulated maternal stress response system has been implicated in the epigenetic transmission of adverse mental health, cognitive, and behavioral outcomes in their children [17]. Moreover, it is not just prenatal exposure but the culmination of stress across a woman’s entire developmental trajectory that may confer risk for poor health in their children [18]. While much of the intergenerational literature is based on animal studies, the findings are robust across species with mechanisms similar to those observed in humans. This evidence of intergenerational transmission of disease risk makes plausible the extended effects of the social, economic, and physical environments. Policy impacts on these environments likely extend much further into the future than previously understood. This is particularly true when considering policies that affect individuals in their reproductive years. To illustrate potential intergenerational implications, we synthesized evidence related to policies from three domains: family leave, nutrition, and housing. We selected these policy domains because they represent increasing distance from a reproductive health focus, each with a growing evidence base to support a potential impact on pregnant women. We reviewed evidence of the impacts of each policy on the health of the offspring.

## 2. Paid Family Leave

Paid family leave (PFL) policies have the most direct implications for intergenerational health, but the full extent has not yet been described. PFL policies have been associated with decreased job turnover [19] and improved maternal mental health in the months and years after a birth [20,21]. Evidence suggests that access to PFL may affect mothers’ mental health trajectories over decades; one recent study found reduced depressive symptoms in late life among women who had access to paid leave around the time of their first birth [22]. The children of adults with access to PFL also benefit, and a substantial body of literature supports the link between PFL and short-term health and developmental outcomes. PFL has been associated with increased breastfeeding initiation and duration [23,24,25], increased immunization rates [26,27], lower infant and child mortality [28,29,30], and higher quality of mother-infant interactions and attachment security [31].

Applying a DOHaD lens, the impact of PFL can be extended in at least two ways. Emerging evidence suggests that PFL may decrease stress among pregnant women—either through offering an opportunity to stop working during pregnancy (antenatal leave) or through the anticipation of paid postpartum leave—and this may result in more appropriate birthweight and decreased PTB [26,28]. Appropriate birth weight is an indicator of fetal developmental processes that contribute to the long-term development of coronary heart disease, diabetes mellitus, and other conditions [32,33,34,35], thus amplifying the potential impact of PFL. The influence of PFL may be further amplified through its well-established connection to breastfeeding. Breastfeeding may provide a link to later life obesity and cardiometabolic disease through healthier infant weight gain [36]. A recent study examined health outcomes among elementary-school-aged children whose parents were exposed to California’s PFL law [37]; the researchers found reduced likelihood of overweight, attention-deficit/hyperactivity disorder (ADHD), hearing problems and frequent ear infections and posit that this likely derives from increased breastfeeding rates. Moreover, in low resource settings, breastfeeding likely accounts for much of the observed link between PFL and infant and child mortality [30,38].

## 3. Sugar Sweetened Beverage (SSB) Taxes

Policies seeking to alter dietary behavior have emerged as promising strategies for improving population health [39]. Sugar sweetened beverage (SSB) purchases are particularly responsive to price changes and, as a result, are a focus of recent policy strategies. Evaluations of existing SSB tax policies suggest that they are effective for reducing SSB purchases and, potentially, SSB consumption, particularly in lower income households. The U.S.’s first major SSB tax was implemented in Berkeley, California in 2015; after 1 year, sales of SSBs declined, while sales of untaxed beverages (e.g., water) increased [40]. While self-reported SSB intake did not change in a representative community sample [40], it decreased in low-income neighborhoods [41]. Two years after Mexico’s 1 peso per liter SSB tax was implemented in 2014, SSB purchases decreased by 9.7%, with greater decreases in lower income households [42].

Simulation studies, which use statistical modeling to predict policy outcomes under differing scenarios, provide information about expected longer-term impacts of SSB tax policies. Several SSB tax simulations have been developed [43,44,45,46,47], each drawing from a vast body of evidence for (1) baseline consumption levels within subgroups; (2) the impacts of SSB price on SSB consumption; and (3) the impacts of SSB consumption on health. First, SSB consumption is high in all age groups, providing a large target population; consumption is highest in adolescents (149–266 kcal/day) and in young to middle-aged adults (151–273 kcal/day) [48]. Second, for every 20% increase in price, consumption decreases by an estimated 24% [49]. Third, greater SSB consumption is associated with higher obesity and diabetes risk in children and adults. For example, a one serving per day increase in SSB consumption is associated with a 15% greater risk of type 2 diabetes [50]. Simulations building from observed reductions in SSB consumption following Mexico’s SSB tax predict a 2.5% reduction in obesity prevalence after 10 years, and prevention of at least 86,000 cases of diabetes by 2030 [51]. The United Kingdom’s tiered levy, passed in 2016, is predicted to reduce obesity prevalence by up to 0.9%, and type 2 diabetes incidence by 31.1 cases per 100,000 person-years [43].

Growing evidence pertaining to prenatal exposure to SSBs offers an opportunity to incorporate intergenerational impacts into policy simulations, from birth outcomes to childhood adiposity. For example, SSBs were the largest single dietary sources of energy consumed in pregnancy (5.6% overall, 8.7% in non-Hispanic Black women) [52]. Observational evidence suggests that greater maternal SSB consumption in pregnancy is associated with adverse birth outcomes associated with life course disease risk in the offspring, including higher risk of PTB [53,54]. Prenatal SSB consumption is also associated with greater adiposity in children ranging from 6 to 8 years of age [55,56]. Epidemiologic evidence is further supported by animal and clinical evidence that maternal fructose consumption alters cardiometabolic processes in the offspring [57], although artificially sweetened beverages may also have detrimental effects [58]. Applying a DOHaD lens to evaluate SSB tax policies requires integration of pregnant women as a subgroup of interest, and extending policy simulations to consider intergenerational effects. It also requires more empirical research on the intergenerational effects of consuming SSBs and their alternatives prior to, during, and after pregnancy, as well as consumption by fathers.

## 4. Housing Policies

Urban renewal policies are seemingly distal from health but when they result in neighborhood gentrification or forced housing transitions, their health effects become apparent. Housing itself is recognized as a social determinant of health, with a large literature providing evidence for its influence [59,60,61]. A recent review by Vasquez-Vera that included 13 high-quality cohort studies, found that just the risk of home foreclosure or the threat of eviction was associated with negative health outcomes, both mental (depression, anxiety, psychological distress, and suicides) and physical (poor self-reported health, high blood pressure, and child maltreatment) [62]. Other work has begun to explore the effects of specific types of housing interventions (e.g., housing subsidies or vouchers) and health outcomes like asthma or binge drinking [8,9,63]. While instructive, the literature rarely extends to the potential effects on pregnancy.

Within this literature exploring the association of housing with pregnancy outcomes, Kramer and colleagues found that women exposed to housing transitions, defined as moving out of public housing, experienced increased risk for preterm LBW but not small for gestational age LBW [64]. Housing instability, defined as two or more moves in one year, has also been associated with increased risk of LBW among young, urban pregnant women [65]. Importantly, the effects of renewal policies that result in housing transitions are differential by race/ethnicity. Recent work exploring the effects of neighborhood gentrification found that among non-Hispanic blacks, very high levels of gentrification (defined as an increase in residents with a college education, higher median household incomes, and a decrease in residents living below the poverty line) was associated with increased PTB compared to non-Hispanic blacks exposed to low levels of neighborhood gentrification. Among non-Hispanic whites, however, living in a very highly gentrified neighborhood was protective against PTB compared to living in a less gentrified neighborhood [66]. These findings are important because the non-white women for whom gentrification is associated with the greatest risk for adverse pregnancy outcomes are the same women whose neighborhoods are most likely to be the targets of urban renewal and neighborhood gentrification [67], thereby all but ensuring an increase in adverse reproductive outcomes.

While the housing-related literature is beginning to concern itself with effects on pregnant women, the consideration of potential health effects stops at birth and fails to take into account the long-term health trajectory that birth outcomes set in motion [34,35,68]. Reviewing urban renewal (e.g., gentrification-supportive) policies through a DOHaD lens would not only require us to consider the positive and negative consequences of what might seemingly be economic development policies, but also the potential effects of these policies on the second generation. From the DOHaD perspective, urban renewal that results in gentrification or resident displacement will likely affect the offspring of pregnant women by increasing the likelihood that these offspring are born too soon (preterm) and/or too small (LBW), both of which set infants on a trajectory for poor adult health [32,34,35].

## 5. Conclusions

Each of these examples represents an opportunity to extend our understanding of policy impact using a DOHaD lens, taking into account the potential life course and intergenerational effects that have previously been overlooked. Increasingly, the policy decision-making process includes efforts to examine, clarify, and estimate the health impacts that policies will have in the short and long term. One specific example of this trend is the health impact assessment (HIA), a valuable tool for (a) systematically evaluating public health impacts of policies and (b) providing recommendations to decision makers as they consider policy adoption and implementation [69]. Here we suggest an analogous process be considered, whereby a DOHaD lens be applied when elucidating the potential impacts of policies that directly impact women of reproductive age.

Consider our first case example, paid family leave. While strong evidence demonstrates PFL impacts across the life course, the adoption of a DOHaD lens allows us to consider intergenerational impacts that extend even further. Specifically, pregnant women who have access to PFL may experience reduced psychological and physiological stress, which in turn may facilitate both improved birth outcomes and women’s ability to initiate and sustain breastfeeding [20,21,24,25,28]. These benefits do not require a DOHaD lens to understand; but when we apply a developmental origins perspective and consider the long-term health impacts of a healthy birthweight, for example, we extend our understanding of the positive intergenerational impacts of PFL.

Similarly, we explicitly connected each of the remaining policy examples (SSB taxes and housing policies) to birth outcomes, which we now understand to shape long-term health trajectories over a life course [32,34,35]. We selected these three cases intentionally, as they differ in focus but share a strong potential for impacting pregnant women supported by an increasing body of evidence. By presenting these three distinct policy areas we have demonstrated—using the DOHaD framework—that true impacts across disparate policies are potentially far greater than we have previously considered.

The DOHaD lens can be applied to numerous other domains. For example, the physical/chemical environment has well-described intergenerational effects and is directly tied to environmental regulations and policy. Persistent organic pollutants, like endocrine disruptors (found in industrial compounds and pesticides) and heavy metals (both naturally-occurring and those resulting from manufacturing and fertilization) are among the most common chemicals found in the environment [70]. These pollutants perturb both male and female urogenital and reproductive systems [71], but also can be found in the cord blood and meconium of exposed offspring [70]. Evidence for epigenetic alterations have also been observed [72]. Exposure to air pollutants, like particulate matter (PM_2.5_) and diesel exhaust, has also been associated with epigenetic changes and adverse outcomes among offspring exposed in utero [73,74,75]. As such, air quality standards and other policies aimed at reducing adverse environmental exposures have a well-documented impact on intergenerational health.

A critical challenge of applying a DOHaD lens to social policy is the clear potential for unintended harm, particularly to women in the form of mother-blame [76]. Application of the DOHaD framework, with its emphasis on gestation and the uterine environment, could inadvertently result in excessive attention to women’s individual-level behaviors and choices, instead of directing our focus where it should be: on developing and sustaining environments that promote health across multiple generations. To avoid this misuse of the DOHaD lens, researchers and policy advocates must be cognizant of the language and imagery they use in order to appropriately frame the issues as being rooted in systems, rather than mothers [76]. By focusing our efforts on macro-level factors and systemic influences, we further discourage efforts that prioritize fetal over maternal benefit—for example, policies that limit women’s agency with the goal of protecting a fetus (e.g., mandated pregnancy leave aimed at limiting exertion). Moreover, given the role of maternal early-life adversity and trauma in the progeneration of vulnerabilities in subsequent generations [77,78], our applications of the DOHaD lens must extend beyond the milieu of pregnancy. As such, our policies should target systemic factors that cause harm to all who experience them, such as poverty, underemployment, and job insecurity; a lack of pregnancy accommodations in the workplace; and lack of control over one’s work environment.

Our analysis further suggests that the adoption of a DOHaD lens may serve to enhance our accuracy in estimating long-term policy effects. One example is through the integration of DOHaD research into policy simulation models. Policy simulations are powerful tools for quantifying and communicating the expected long-term health (and other) benefits of policies, thereby supporting policy decision-making. The accuracy of these models, however, depends on the quality of underlying information inputs; incorporating research on the links between birthweight and life course trajectories, for example, may improve the accuracy of these predictions. Future studies using policy simulation models should incorporate data on life course trajectories to directly examine these long-term effects.

We suggest that incorporating DOHaD into our assessment encourages consideration of policy impacts on critical periods (e.g., early childhood, school entry, adolescence), both independently and synergistically with prenatal exposures, which have far-reaching implications for population health. That is, prenatal exposure comprises a “first hit”, priming the offspring with heightened susceptibility to adverse health; postnatal exposure then presents a “second hit”, through which prenatally primed susceptibility can be triggered or exacerbated. This magnifies the potential impact of a policy if we consider both the prevention of a first hit and mitigation of second hits.

We described three policies that illustrate the potential utility of applying a DOHaD lens, but we could have selected from among many others. We hope that this work stimulates others to conduct theoretical and empirical studies of the life course and intergenerational impacts of diverse policy areas. In particular, future studies could attempt to empirically stitch together existing evidence on the health impacts of a policy across generations to estimate the surplus impact not previously reported in the literature. Endeavors to assess health effects of policy decisions, particularly those targeting women of reproductive age, will be enhanced by applying a DOHaD lens to ensure full consideration of the potential impact on future generations.
